# Ejaculation-preserving holmium laser enucleation of the prostate: a systematic review of techniques, functional outcomes, and safety

**DOI:** 10.1007/s00345-026-06348-7

**Published:** 2026-03-21

**Authors:** Mehmet Hamza Gultekin, Kadir Can Sahin, Admir Ozturk, Feyyaz Irmak, Bulent Onal

**Affiliations:** 1 Cerrahpasa Faculty of Medicine, Department of Urology, Istanbul University – Cerrahpasa, Koca Mustafapasa Cd. No:53, 34098 Fatih, Istanbul, Turkey; 2 Department of Urology, Istanbul Bayrampasa State Hospital, Istanbul , Turkey; 3 Cerrahpasa Faculty of Medicine, Istanbul University – Cerrahpasa, Istanbul , Turkey

**Keywords:** Benign Prostatic Hyperplasia, Ejaculation, Ejaculatory Dysfunction, Holmium Laser Enucleation of the Prostate, Lower Urinary Tract Symptoms, Prostatic Diseases, Systematic Review

## Abstract

**Context:**

Holmium laser enucleation of the prostate (HoLEP) is a guideline-endorsed, size-independent surgical standard for benign prostatic hyperplasia (BPH). However, conventional HoLEP is frequently associated with loss of antegrade ejaculation (AE), an outcome of increasing importance for sexually active patients. Anatomically informed modifications aimed at preserving peri-ejaculatory structures have been proposed, yet the evidence supporting antegrade ejaculation-preserving HoLEP (EP-HoLEP) remains fragmented.

**Objective:**

To systematically review and synthesize the contemporary evidence on ejaculation-preserving HoLEP techniques, focusing on ejaculatory outcomes, urinary function, and surgical safety.

**Evidence Acquisition:**

A systematic literature search of PubMed and Embase was conducted in accordance with the PRISMA 2020 guidelines to identify studies evaluating EP-HoLEP techniques published through December 2025. Randomized controlled trials, prospective comparative studies, and retrospective cohorts reporting ejaculatory outcomes were eligible. Risk of bias was assessed using RoB 2, ROBINS-I, and MINORS tools as appropriate.

**Evidence Synthesis:**

Ten studies encompassing 1,675 patients were included, comprising three randomized controlled trials, four non-randomized comparative studies, and three single-arm case series. EP-HoLEP techniques were categorized as ejaculatory hood–sparing, mucosal and sphincter-sparing (including modified two-lobe and Double-n techniques), and selective median-lobe enucleation. Hood-sparing approaches alone demonstrated limited efficacy in preserving AE. In contrast, techniques that preserved peri-verumontanum tissue, anterior urethral mucosa, and bladder-neck fibers consistently achieved higher AE preservation rates, reaching 70–85% in selected cohorts. Selective median-lobe enucleation yielded the highest AE preservation in anatomically favourable patients but was associated with less pronounced symptom relief in some series. Across studies, improvements in International Prostate Symptom Score and maximum urinary flow rate were comparable to standard HoLEP, with no consistent increase in perioperative complications, although some techniques required longer operative times. Overall methodological quality was moderate, with residual confounding and heterogeneity limiting direct comparison.

**Conclusions:**

EP-HoLEP techniques can substantially improve rates of antegrade ejaculation without compromising short-term urinary outcomes in appropriately selected patients. Effective preservation appears to depend on anatomically informed modification of the enucleation plane rather than superficial tissue sparing alone. EP-HoLEP represents a spectrum of techniques rather than a single standardized approach, and potential trade-offs related to operative complexity and durability should be discussed during patient counselling. High-quality, standardized prospective studies are needed to define optimal techniques and long-term outcomes.

**Patient Summary:**

Modified HoLEP techniques that preserve key anatomical structures involved in ejaculation may help sexually active men maintain normal ejaculation while still achieving effective relief of urinary symptoms from prostate enlargement.

**Supplementary Information:**

The online version contains supplementary material available at 10.1007/s00345-026-06348-7.

## Introduction

Benign prostatic hyperplasia (BPH) is a highly prevalent condition in aging men and remains a major cause of lower urinary tract symptoms (LUTS) [1]. Holmium laser enucleation of the prostate (HoLEP) has evolved over the last two decades into a size-independent, guideline-endorsed surgical standard for BPH, offering durable symptom relief and low retreatment rates [2].

Despite these advantages, conventional HoLEP, like classic transurethral resection of the prostate (TURP), is frequently associated with loss of antegrade ejaculation (AE), with reported rates of retrograde or absent ejaculation approaching 70–80% in sexually active men [3]. As BPH surgery increasingly targets younger, sexually active patients, preservation of ejaculatory function has become a critical outcome alongside relief of obstruction.

The physiology of ejaculation is complex and involves coordinated emission and expulsion phases regulated by sympathetic and somatic pathways [4]. Traditionally, retrograde ejaculation (RE) following prostate surgery has been attributed to impaired bladder neck closure [5]. However, anatomical and imaging studies increasingly suggest that structures surrounding the verumontanum and proximal urethra, rather than the bladder neck alone, play a critical role in directing ejaculate antegradely [6–8]. These insights have led to a new paradigm in which preservation of peri-verumontanum tissue, intraprostatic urethral mucosa, and bladder-neck structures, rather than mere maintenance of bladder neck competence, is considered crucial for maintaining AE. In support of this concept, Sturch et al. proposed that much of the RE observed after BPH surgery may reflect disruption of this high-pressure ejaculatory zone rather than unavoidable reflux through an incompetent bladder neck [5].

Building on these concepts, tissue-sparing principles have motivated the development of ejaculation-preserving modifications of endoscopic BPH surgery, including early adaptations of TURP and laser-based procedures [9, 10], raising the question of whether similar preservation strategies could be adapted to HoLEP. Applying antegrade ejaculation-preserving concepts to HoLEP is more challenging, because classical three-lobe or en-bloc HoLEP achieve complete adenoma enucleation along the surgical capsule, potentially sacrificing peri-verumontanum and paracollicular structures relevant to ejaculation [11].

**Fig. 1:**
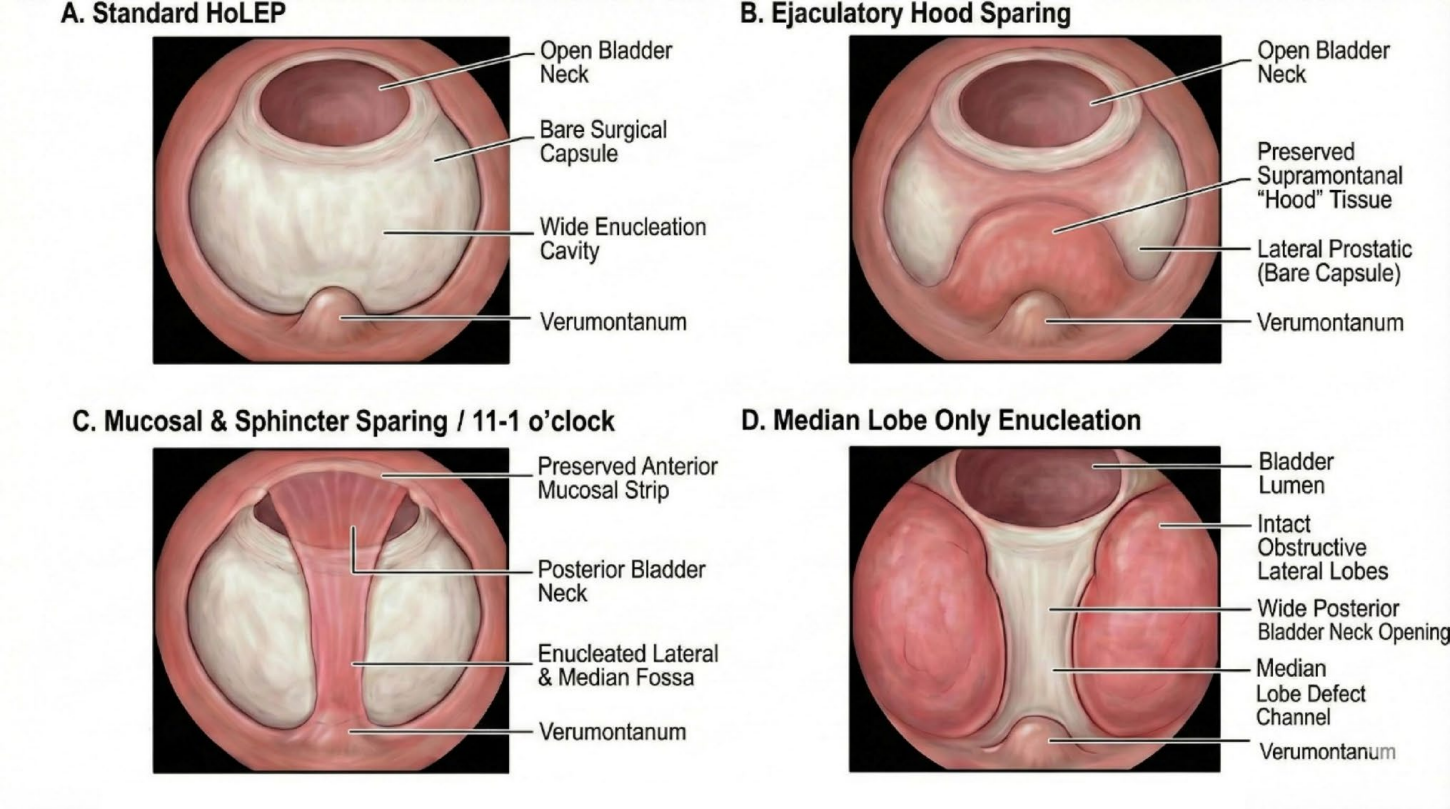
Schematic representation of standard and ejaculation-preserving HoLEP techniques. **A** Standard HoLEP, demonstrating complete adenoma enucleation with exposure of the surgical capsule, a widely open bladder neck, and a large enucleation cavity extending to the verumontanum. **B** Ejaculatory hood–sparing HoLEP, in which supramontanal (“ejaculatory hood”) tissue is preserved while lateral adenomatous tissue is enucleated. **C** Mucosal and sphincter-sparing HoLEP (11–1 o’clock preservation), illustrating preservation of the anterior urethral mucosal strip and peri-sphincteric tissue, with enucleation of lateral and median adenoma while maintaining posterior bladder neck continuity. **D** Selective median-lobe enucleation, showing isolated removal of the obstructing median lobe with preservation of the lateral lobes and maintenance of a wide posterior bladder neck channel, leaving the peri-verumontanum anatomy intact

Nevertheless, various surgical adaptations have been proposed to better respect peri-ejaculatory anatomy while preserving the fundamental principles of anatomical enucleation. These adaptations include hood-sparing or apical mucosa–sparing HoLEP, modified two-lobe techniques that preserve urethral mucosa and bladder-neck tissue, en-bloc HoLEP with targeted bladder-neck preservation, urethral ridge or urethral mucosa preservation from bladder neck to apex, and selective median-lobe HoLEP in anatomically favourable prostates (Fig. 1). As these approaches have expanded, a heterogeneous spectrum of techniques has emerged, each differing in dissection plane, the extent of tissue preservation, and its theoretical impact on ejaculatory function.

Despite this growing interest, the evidence base for antegrade ejaculation-preserving HoLEP (EP-HoLEP) remains fragmented. Existing studies vary in design, patient selection, definitions of “ejaculation preservation,” and the consistency of urinary and sexual outcome reporting, limiting direct comparison across techniques. This heterogeneity complicates interpretation of benefits, risks, and potential trade-offs related to operative efficiency, completeness of adenoma removal, and long-term functional outcomes To date, no systematic review has synthesized the contemporary literature on ejaculation-preserving HoLEP, evaluated the spectrum of proposed techniques, or critically examined their implications for ejaculatory function, urinary outcomes, and surgical safety.

We therefore performed a systematic review of EP-HoLEP techniques in sexually active men with BPH. The primary aim was to compare preservation of AE between EP-HoLEP and standard techniques, with secondary aims evaluating urinary function, continence status, peri-operative safety, and the need for reintervention across different preservation approaches.

## Materials and methods

### Search strategy and study design

This systematic review was conducted in strict adherence to the Preferred Reporting Items for Systematic Reviews and Meta-Analyses (PRISMA 2020) guidelines [12]. A comprehensive and systematic search of the literature was performed using the PubMed and Embase databases to identify relevant studies published up to December 2025. The search strategy was designed to capture all studies evaluating ejaculation-sparing modifications of HoLEP. To ensure maximum sensitivity, the search string incorporated a combination of Medical Subject Headings (MeSH) and free-text terms. The complete electronic search strategies are provided in Supplementary Table S1 to ensure transparency and reproducibility. No restrictions regarding language or publication date were applied during the initial search process.

### Eligibility criteria

Studies were selected based on predefined inclusion criteria framed by the PICO (Population, Intervention, Comparison, Outcome) model.

*Population (P):* Adult male patients diagnosed with BPH undergoing surgical treatment.

*Intervention (I):* HoLEP incorporating technical modifications specifically intended to preserve AE, including but not limited to ejaculatory hood–sparing, selective median-lobe enucleation, urethral mucosa–sparing, and Double-n techniques.

*Comparator (C):* Standard or conventional HoLEP, other surgical modalities (e.g., TURP), or preoperative baseline status in single-arm studies where no formal comparator group was available.

*Outcomes (O):* Studies had to report quantitative or qualitative ejaculatory function outcomes, including rates of preserved AE, incidence of RE, or validated sexual function measures such as the MSHQ-EjD(-SF). Secondary outcomes, when available, included urinary symptoms (IPSS), maximum urinary flow rate (Qmax), continence, perioperative outcomes, and retreatment.

Eligible study designs included randomized controlled trials (RCTs), prospective comparative studies, and retrospective cohort studies.

Records were excluded if they were conference abstracts, editorials, expert opinions, or review articles. Additionally, clinical trial protocols with no posted results, studies utilizing non-enucleation laser techniques (e.g., vaporization, Greenlight, Diode), and studies failing to report specific ejaculatory data were excluded from the analysis.

### Study selection and data extraction

The study selection process was executed in two consecutive stages. Initially, titles and abstracts were screened for relevance to the study topic. Subsequently, full-text articles of potentially eligible studies were retrieved and assessed against the inclusion criteria by two independent reviewers. Any discrepancies regarding study eligibility were resolved through consensus.

**Fig. 2:**
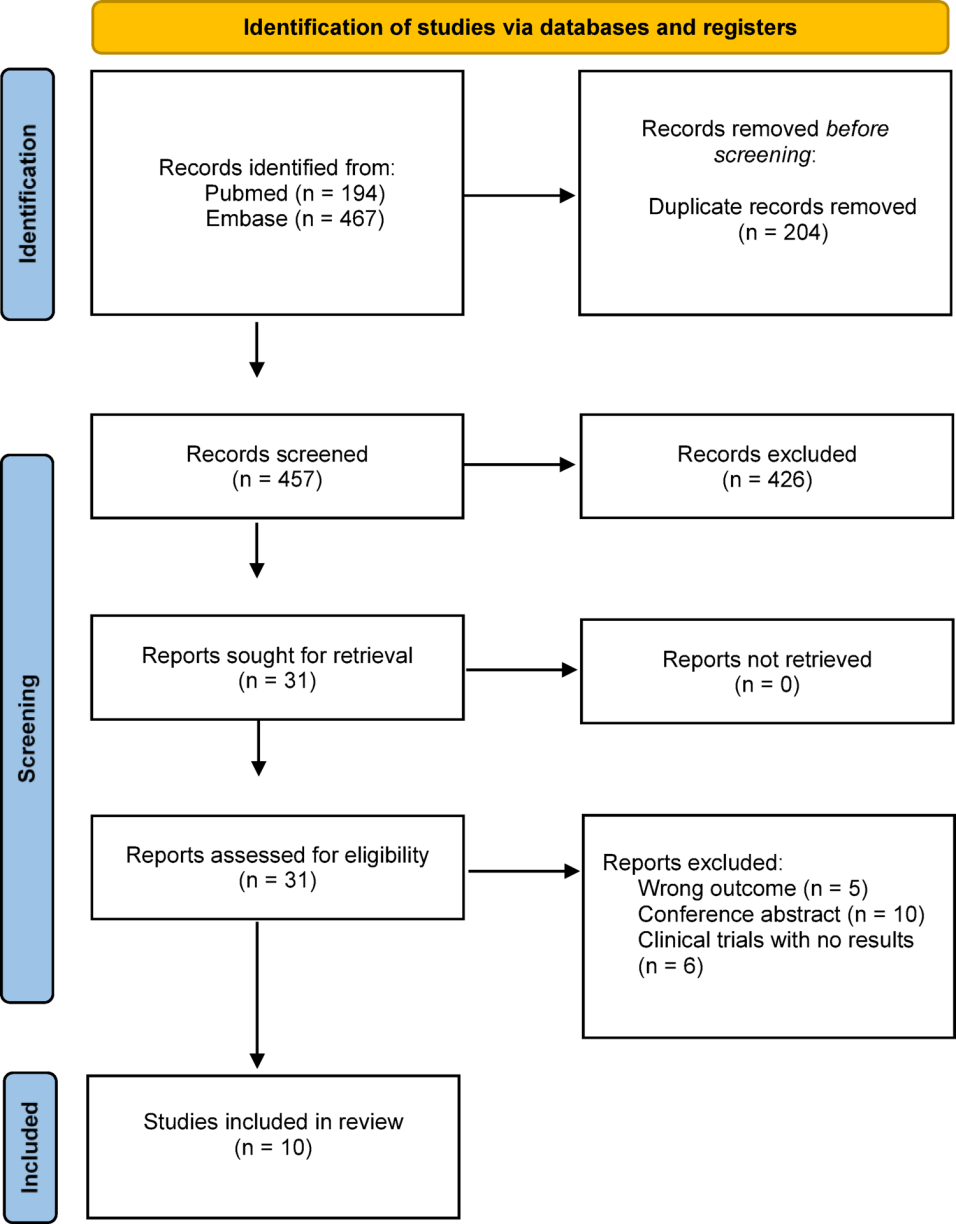
Flow diagram of study identification, screening, eligibility assessment, and inclusion in the systematic review, in accordance with the PRISMA 2020 guidelines

The initial comprehensive literature search yielded a total of 661 citations. Following the removal of 204 duplicate records, 457 unique citations remained for the title and abstract screening phase. Of these, 426 records were excluded as they did not meet the inclusion criteria or were deemed irrelevant. Consequently, 31 full-text articles were retrieved and assessed for eligibility. Upon detailed review, 21 articles were excluded for specific reasons: 10 were conference abstracts lacking full text, 6 were clinical trial protocols with no posted results, and 5 studies did not report specific data on ejaculation outcomes. Ultimately, 10 studies met all eligibility criteria and were included in the systematic review (Fig. 2). Given the substantial heterogeneity in study design, surgical techniques, outcome definitions, and follow-up duration, a quantitative meta-analysis was not planned a priori.

Data were extracted using a standardized extraction form, capturing key study characteristics including author, publication year, study design, population demographics (sample size, age, prostate volume), detailed descriptions of the surgical technique, and clinical outcomes including ejaculation preservation rates, International Prostate Symptom Scores (IPSS), maximum urinary flow rate (Qmax), and perioperative complications.

This systematic review was prospectively registered in the International Prospective Register of Systematic Reviews (PROSPERO; registration number CRD420251274645).

### Quality assessment

The methodological quality and potential risk of bias were evaluated using tools tailored to the specific design of the included studies. Randomized controlled trials were assessed using the Cochrane Risk of Bias 2 (RoB 2) tool [13]. For non-randomized comparative studies, the updated ROBINS-I V2 (Risk Of Bias In Non-randomized Studies - of Interventions) tool was utilized to assess the risk of confounding and selection bias [14]. Non-comparative (single-arm) studies were evaluated using the MINORS (Methodological Index for Non-Randomized Studies) tool, as standard comparative bias tools were not applicable to these designs [15].

## Results

The included literature was published between 2014 and 2025, comprising a pooled population of 1,675 male patients diagnosed with BPH. The study designs included three RCTs, four non-randomized comparative studies, and three retrospective single-arm case series. The surgical interventions were categorized into three primary modifications: ejaculatory hood sparing, mucosal and sphincter sparing (including Double-n and Urethral Ridge techniques), and selective median lobe enucleation. Detailed characteristics of the included studies are provided in Table 1 [16–25].

**Table 1:** Study characteristics of the included EP-HOLEP studies, including study design, patient population, prostate characteristics, surgical technique, comparator (if applicable), follow-up duration, and reported urinary and ejaculatory outcomes

Study (Author, Year)	Study Design	Sample Size (*N*) & Groups	Surgical Intervention (Technique)	Comparator	Primary Outcomes (Ejaculatory Function)	Secondary Outcomes (Urinary Function & Safety)	Follow-up Duration
Kim et al. (2014) [16]	Prospective Pilot Study (Non-RCT)	*N* = 52 • EP-HoLEP: 26 • Conventional: 26	**Ejaculatory Hood Sparing**: Preservation of paracollicular and supracollicular tissue > 1 cm proximal to the verumontanum.	Conventional HoLEP (Standard 3-Lobe)	EP rate: • EP-HoLEP: 46.2% • Conventional: 26.9% *(p = 0.249)*	No significant difference in IPSS, Qmax, or PVR between groups. Sparing only the hood is insufficient without apical tissue preservation.	9.7 months (mean)
Xu et al. (2019) [17]	RCT	*N* = 191 • Modified: 97 • Traditional: 94	**Modified Two-Lobe**: Preservation of the urethral membrane between 11 and 1 o’clock positions and bladder neck mucosa.	Conventional HoLEP (3-Lobe)	RE rate at 12 months: • Modified: **13.33%** • Traditional: 50.0% *(p = 0.034)*	UI rate was significantly lower in the modified group (1.03% vs. 8.51%, *p* = 0.036). IPSS and QoL improved in both.	12 months
Rouf et al. (2021) [18]	Prospective comparative cohort	*N* = 119 • EP-HoLEP: 63 • EP-TURP: 56	**EP-HoLEP**: Preservation of paracollicular tissue and area 1 cm proximal to verumontanum.	EP-TURP	AE preservation: • EP-HoLEP: **22.1%** • EP-TURP: 85.7%	Significant improvement in Qmax and IPSS in both groups. However, sexual satisfaction significantly deteriorated in the EP-HoLEP group compared to EP-TURP.	6 months
Li et al. (2021) [19]	Retrospective cohort	*N* = 704 (Total) *n* = 213 (Sexual function analysis)	**En-bloc + Bladder Neck Preservation**: 0.5 cm of bladder neck tissue preserved; en-bloc resection with early release.	None (Single-arm analysis)	Post-op RE rate: **11.7%** (25/213 patients). Semen volume: • Unchanged: 56.4% • Decreased: 43.6%	Significant improvement in Qmax, IPSS, and PVR (*p* < 0.05). Low incontinence rate (5.4% mild, 0.4% moderate immediately post-op).	12 months
Press et al. (2023) [20]	Retrospective case series	*N* = 55 (40 sexually active)	**Median Lobe Only**: Selective enucleation of the median lobe; lateral lobes and apical tissue completely preserved.	None (Case series)	EP rate: **87.5%** (35/40 men reported normal AE). New dysfunction: 5% (2/40)	IPSS improved from 22.5 to 6.9 (*p* < 0.001). No patients reported stress urinary incontinence.	4.7 months (mean)
Long Depaquit et al. (2024) [21]	Retrospective cohort	*N* = 55	**ML-HOLEP (Median Lobe Only)**: Selective laser enucleation of the median lobe.	None (Single-arm)	De novo RE rate: **12.5%** (4/32 patients with pre-operative AE).	Significant improvement in IPSS and Qmax. However, 14.5% required surgical reintervention due to persistent LUTS.	12 months
Qiu et al. (2024) [22]	Retrospective comparative	*N* = 208 • Observation: 113 • Control: 95	**Urethral Mucosa Preservation**: Preservation of the longitudinal urethral mucosa from the bladder neck to the prostate tip.	Conventional HoLEP (No preservation)	RE incidence: • Observation: **13.27%** • Control: 25.26% *(p < 0.05)*	Immediate urinary continence rate was higher in the observation group (98.23% vs. 85.26%, *p* < 0.05).	Not mentioned
Fang et al. (2024) [23]	Retrospective comparative	*N* = 80 • Modified: 30 • Control: 50	**Urethral Ridge Preservation**: Preservation of the mucosa at the 12 o’clock groove (“urethral ridge”) for approx. 0.5 cm.	Conventional HOLEP	RE incidence: • Modified: **16.7%** • Control: 38.0% *(p < 0.05)*	Immediate urinary control rate was significantly better in the modified group (93.3% vs. 76%, *p* < 0.05).	6 months
Eliwa et al. (2025) [24]	RCT	*N* = 43 • EP-HOLEP: 21 • Standard: 22	**EP-HOLEP**: Preservation of 10 mm supramontanal tissue + 5 mm paracollicular tissue + Mucosal strip at 2 & 10 o’clock.	Conventional HoLEP	MSHQ-EjD-SF scores: Significantly higher in the EP-HoLEP group at 3 and 6 months post-operatively *p < 0.01)*.	Incontinence episodes (ICIQ-UI SF) were significantly lower in the EP group at 1 month (*p < 0.023*). IPSS improvement was comparable. ^9^	6 months
Gao et al. (2025) [25]	RCT	*N* = 128 (Analyzed) • Group A (Std): 39 • Group B (Single-n): 44 • Group C (Double-n): 45	**Double-n Technique**: Preservation of urethral mucosa at 11 and 1 o’clock positions (inverted U-shape) and paracollicular tissue.	• Group A: Standard • Group B: Single-n (Supramontanal only)	AE rate (12 months): • Double-n (Group C): **77.8%** • Single-n (Group B): 45.5% • Standard (Group A): 23.1% *(p < 0.05)*	Qmax and IPSS improved similarly across all groups. Operative time was significantly longer in the Double-n group (*p < 0.05*).	12 months

*RCT*; randomized controlled trial, *HoLEP*; Holmium laser enucleation of the prostate, *TURP*; transurethral resection of the prostate, *EP*; ejaculation preserving, *IPSS*; international prostate symptom score, *Qmax*; maximum urinary flow rate, *PVR*; post-voiding residual urine, *AE*; antegrade ejaculation, *RE*; retrograde ejaculation, *MSHQ-EjD-SF*; Male Sexual Health Questionnaire Ejaculatory Dysfunction, *ICIQ-UI SF*; International Consultation on Incontinence Questionnaire Short Form.

### Risk of Bias and Methodological Quality

**Fig. 3:**
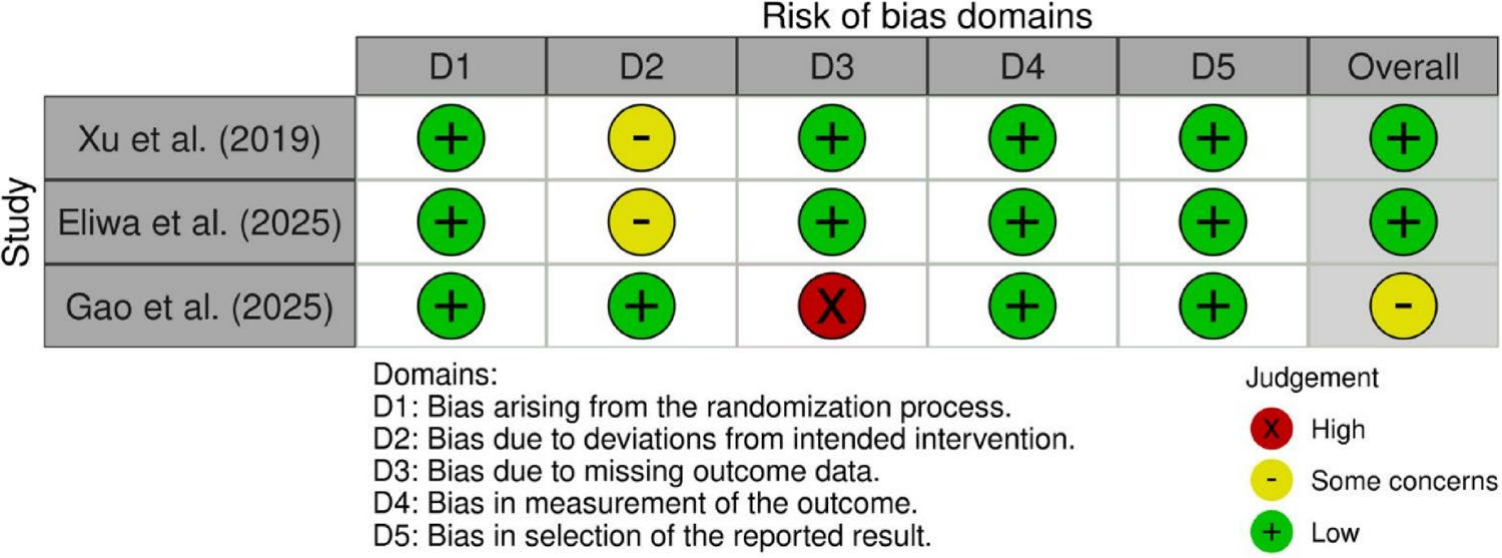
Risk of bias assessment of randomized controlled trials included in the review, evaluated using the Cochrane Risk of Bias 2 (RoB 2) tool across the five standard domains. Overall judgements reflect concerns primarily related to deviations from intended interventions and outcome measurement

**Fig. 4:**
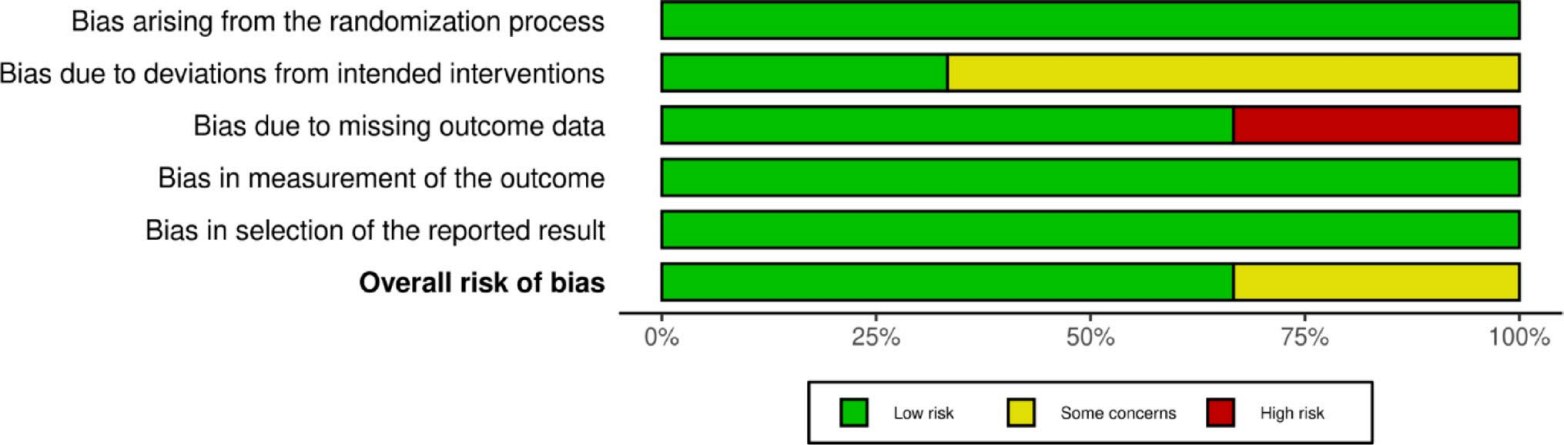
Risk of bias summary for randomized controlled trials assessed using the Cochrane Risk of Bias 2 (RoB 2) tool. The figure illustrates the proportion of included randomized controlled trials judged as having low risk of bias, some concerns, or high risk of bias across the five RoB 2 domains: randomization process, deviations from intended interventions, missing outcome data, measurement of the outcome, and selection of the reported result. The overall risk of bias reflects the aggregate judgement across all domains for each trial

**Fig. 5:**
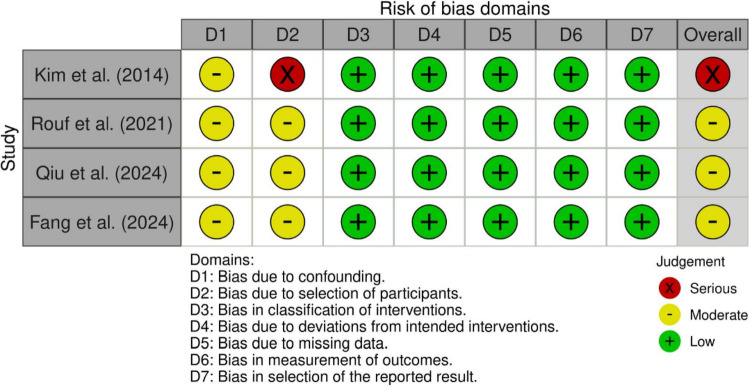
Risk of bias assessment of non-randomized comparative studies included in the review, evaluated using the ROBINS-I tool across domains including confounding, selection of participants, classification of interventions, deviations from intended interventions, missing data, outcome measurement, and selective reporting. Green indicates low risk of bias, yellow indicates moderate risk or some concerns, and red indicates serious risk of bias

**Fig. 6:**
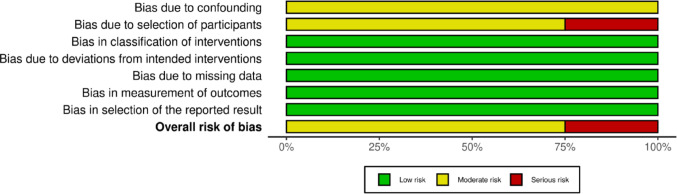
Risk of bias summary for non-randomized comparative studies assessed using the ROBINS-I tool. The figure presents the proportion of included non-randomized studies judged as having low, moderate, or serious risk of bias across the seven ROBINS-I domains: bias due to confounding, selection of participants, classification of interventions, deviations from intended interventions, missing data, measurement of outcomes, and selection of the reported result. No study was judged to have a critical risk of bias. The overall risk of bias represents the aggregated judgement across all domains for each study

The methodological quality of the included studies was evaluated using tools appropriate for each study design. The summaries of these assessments are visually presented in Figs. 3, 4, 5 and 6; Table 2.

#### Randomized controlled trials

The risk of bias for the three RCTs was assessed using the Cochrane RoB 2 tool (Figs. 3 and 4). Overall, the quality of the randomized evidence was mixed. Xu et al. and Eliwa et al. were rated as having a “Low” risk of bias overall, although both studies raised “Some concerns” in Domain 2 (Deviations from intended interventions) [17, 24]. This domain was rated as ‘some concerns’, reflecting the inherent challenges of blinding in surgical trials. Conversely, Gao et al. was rated as raising “Some concerns” overall, driven by a “High” risk rating in Domain 3 (Missing outcome data) due to a loss-to-follow-up rate exceeding 20%, which introduces potential attrition bias [25].

**Table 2:** Methodological quality assessment of non-randomized studies included in the review, evaluated using the Methodological Index for Non-Randomized Studies (MINORS) criteria

Study	Aims (0-2)	Inclusion Criteria (0-2)	Prospective Data (0-2)	Endpoints (0-2)	Unbiased Assessment (0-2)	Follow-up Period (0-2)	Loss to Follow-up (0-2)	Prospective Calculation (0-2)	Total Score (x/16)
Li et al. (2021) [19]	2	2	0	2	0	2	2	0	**10** (Moderate)
Press et al. (2023) [20]	2	2	0	2	0	2	2	0	**10** (Moderate)
Depaquit et al. (2024) [21]	2	2	0	2	0	2	2	0	**10** (Moderate)

#### Non-randomized comparative studies

The quality of the four observational comparative studies was evaluated using the standard ROBINS-I tool, as shown in Figs. 5 and 6. Kim et al. was classified as having a “Serious” overall risk of bias [16]. This judgement was primarily influenced by Domain 2 (Bias due to selection of participants), as the study utilized an alternating allocation method rather than true randomization, and Domain 1 (Confounding), where baseline characteristics were not adequately adjusted. The remaining three studies —Rouf et al., Qiu et al., and Fang et al.— were assessed as having a “Moderate” overall risk of bias [18, 22, 23]. While these studies generally demonstrated low risk in domains regarding missing data (D5) and outcome measurement (D6), they consistently showed moderate risk in Domain 1 (Confounding) and Domain 2 (Selection). This is consistent with their retrospective design, where residual confounding from unmeasured variables (such as surgeon preference in patient selection) cannot be entirely excluded.

#### Non-comparative studies

The methodological quality of the three single-arm case series was assessed using the MINORS index (Table 2). The assessment revealed a consistent profile across Li et al., Press et al., and Depaquit et al. [19–21]. All three studies demonstrated “High” risk (Red) in Domain 3 (Prospective collection of data), Domain 5 (Unbiased assessment of the study endpoint), and Domain 8 (Prospective calculation of the study size). These deficits are inherent to retrospective case series designs lacking pre-registered protocols and blinded assessors. However, the studies scored well (“Low” risk) in defining aims, inclusion criteria, and endpoints, resulting in a moderate overall quality score (10/16) for all three. Overall, high-quality randomized evidence supports mucosal- and sphincter-sparing EP-HoLEP techniques, whereas observational data, although suggestive, remain limited by residual confounding Fig. 6.

### Ejaculatory function outcomes

#### Ejaculatory hood sparing techniques

Studies evaluating techniques that focused solely on preserving the supramontanal tissue (the “ejaculatory hood”) without addressing the apical mucosa or bladder neck fibers yielded suboptimal results. Kim et al. reported an AE preservation rate of 46.2% in the sparing group, which was not statistically superior to the 26.9% observed in the conventional HoLEP group (*p* = 0.249) [16]. Similarly, Rouf et al. found that their hood-sparing EP-HoLEP technique resulted in a preservation rate of only 22.1%, which was significantly inferior to the 85.7% preservation rate achieved in the comparative ejaculation-preserving TURP group [18].

#### Mucosal and sphincter sparing techniques

Surgical modifications that extended preservation to include the anterior urethral mucosa (typically between the 11 and 1 o’clock positions), bladder neck fibers, and paracollicular tissue were associated with higher rates of AE in both RCTs and comparative studies. In a randomized trial, Xu et al. reported a significantly reduced RE rate of 13.3% in the modified two-lobe group compared to 50.0% in the traditional three-lobe group (*p* = 0.034) [17]. Validating this approach, Gao et al. demonstrated that the “Double-n” technique achieved a 77.8% AE rate at 12 months, which was statistically superior to both the “Single-n” (45.5%) and standard (23.1%) techniques (*p* < 0.05) [25]. Furthermore, Eliwa et al. observed significantly higher MSHQ-EjD-SF scores in the preservation group compared to standard HoLEP (Median 15 vs. 6, *p* = 0.01) [24]. Retrospective analyses by Li et al., Qiu et al., and Fang et al. corroborated these findings, reporting RE rates between 11.7% and 16.7%, consistently lower than historical controls [19, 22, 23].

#### Selective median lobe enucleation

The approach of selectively enucleating only the obstructing median lobe while sparing the lateral lobes proved highly effective for preservation in anatomically suitable patients. Press et al. reported that 87.5% of sexually active men maintained normal AE following this procedure [20]. Long Depaquit et al. similarly reported a low de novo RE rate of 12.5% [21]. However, this technique highlighted a potential drawback regarding symptom relief in some cohorts.

### Secondary outcomes: urinary function and safety

The adoption of ejaculation-sparing modifications did not compromise the primary surgical goal of relieving bladder outlet obstruction. All included studies reported statistically significant postoperative improvements in IPSS and Qmax compared to baseline (*p* < 0.05). In comparative studies, no statistically significant differences were found in final IPSS scores or Qmax values between the preservation and standard HoLEP groups at six to 12 months follow-up. Additionally, several studies suggested a protective effect on urinary continence. Xu et al. reported a transient urinary incontinence rate of 1.03% in the modified group versus 8.51% in the standard group (*p* = 0.036) [17]. Similarly, Eliwa et al. observed significantly fewer incontinence episodes in the preservation group at 1-month follow-up (*p* = 0.023), and Fang et al. reported a significantly higher immediate urinary control rate in the modified group (93.3%) compared to controls (76.0%) [23, 24]. Regarding safety, no significant differences were reported in perioperative complications such as bleeding or infection, although Gao et al. noted that the “Double-n” technique required a significantly longer operative time (*p* < 0.05) [25].

## Discussion

This systematic review synthesizes the contemporary evidence on EP-HoLEP and demonstrates that selective anatomical preservation during enucleation can meaningfully improve rates of antegrade ejaculation compared with conventional HoLEP, while maintaining clinically relevant relief of lower urinary tract symptoms. Importantly, the reviewed literature indicates that ejaculatory preservation is not a binary outcome but is closely related to the extent and location of tissue spared during enucleation, reinforcing the importance of peri-ejaculatory anatomy in surgical planning. Preservation of sexual function, including ejaculation, is increasingly recognized as a relevant outcome in contemporary BPH management [1].

Traditionally, loss of AE after TURP has been attributed to impaired bladder neck closure. However, accumulating anatomical, histomorphological, and imaging data indicate that ejaculation depends on the coordinated function of peri-verumontanum, intraprostatic urethral, and sphincteric structures rather than bladder neck competence alone [6–8, 26]. This evolving understanding provides the conceptual foundation for contemporary EP-HoLEP strategies that seek to preserve key peri-ejaculatory anatomy during enucleation. These evolving physiological concepts are consistent with recent analyses emphasizing the functional importance of the ejaculatory hood, peri-verumontanal musculature, and sub-sphincteric emission mechanisms in contemporary BPH surgery [27].

The heterogeneity of EP-HoLEP techniques identified in this review reflects differing surgical interpretations of which anatomical elements are most critical for ejaculation, rather than inconsistency in the underlying physiological concept. Early attempts at EP-HoLEP, particularly those limited to superficial ejaculatory hood or paracollicular mucosal sparing, yielded inconsistent results, likely because they preserved visible landmarks without adequately respecting the deeper muscular structures involved in emission and expulsion [16]. These early experiences highlight that preservation confined to the mucosal hood alone is insufficient and that effective ejaculation preservation requires a more anatomically informed modification of the enucleation plane.

More contemporary EP-HoLEP approaches—including modified two-lobe enucleation, urethral mucosa-sparing techniques, targeted bladder-neck preservation, and selective median-lobe enucleation—share a common principle of deliberate avoidance of dissection in regions closely associated with peri-verumontanum and proximal urethral sphincteric anatomy [17, 18, 22]. Rather than abandoning the enucleation concept, these techniques attempt to redefine the safe dissection boundaries within the prostatic urethra. Selective median-lobe HoLEP represents a distinct conceptual strategy, emphasizing anatomical patient selection rather than global modification of the enucleation technique, and illustrates that ejaculation preservation may be achievable by limiting surgical intervention in anatomically favourable prostates [20, 21]. However, such approaches may not be applicable across the full spectrum of prostate morphologies and instead underscore the importance of a patient-based approach that integrates individual anatomical configuration, prostate morphology, and patient priorities when selecting an ejaculation-preserving strategy.

An additional and clinically relevant contributor to this heterogeneity is prostate volume. When prostate volume distributions across the included studies are examined, the available evidence predominantly reflects outcomes in patients with moderate-sized glands, particularly in the 40–80 g range. Notably, only one randomized study explicitly defined a prostate volume inclusion criterion, while most others either did not report volume-based selection or included heterogeneous gland sizes without stratified analysis. This lack of standardized reporting likely contributes to the heterogeneity observed in ejaculatory and functional outcomes across studies and limits the generalizability of ejaculation-preserving HoLEP techniques, particularly to patients with large-volume prostates. In such cases, technical factors—including the feasibility of bladder neck preservation, the extent of tissue sparing, and maintenance of adequate enucleation planes—may differ substantially and influence both ejaculatory and urinary outcomes. Accordingly, prostate volume should be explicitly considered during patient selection and surgical planning, and future studies should incorporate volume-stratified analyses to better define the anatomical boundaries within which ejaculation-preserving HoLEP can be reliably applied.

A patient-based approach is therefore important when considering ejaculation-preserving HoLEP. Suitability for EP strategies depends on individual anatomy, prostate morphology, and patient priorities rather than a uniform set of criteria. From an anatomical standpoint, EP-HoLEP may be most feasible in men with relatively preserved peri-verumontanum and apical anatomy, a clearly identifiable surgical capsule, and prostate configurations that allow controlled dissection without extensive disruption of paracollicular structures. Patients with a dominant intravesical median lobe and limited lateral lobe obstruction may be particularly suitable for selective median-lobe approaches, whereas those with bulky circumferential adenomas or marked apical distortion may be less amenable to highly tissue-sparing techniques. Prostate size may also influence strategy selection; in moderate-sized glands, mucosal- and paracollicular-sparing techniques are often technically more manageable, whereas in very large prostates (e.g., > 100 g) extensive sparing can be challenging and may increase operative complexity. Beyond short-term functional outcomes, the durability of ejaculation-preserving approaches warrants careful consideration. Although these modifications can effectively maintain antegrade ejaculation in selected patients, they may involve important trade-offs that should be addressed during preoperative counseling, particularly the need to balance short-term sexual benefit against potential long-term functional outcomes.

In anatomically favorable prostates—especially those with a dominant intravesical median lobe—selective median-lobe enucleation can provide excellent early preservation of ejaculation and meaningful symptom relief; however, by intentionally narrowing the enucleation field, this approach may leave residual adenomatous tissue and has been associated in some series with higher rates of persistent LUTS and subsequent reintervention (up to 14.5%). Thus, the short-term advantage in ejaculatory preservation may, for a subset of patients, come at the cost of reduced durability of symptom relief.

In prostates without a dominant median lobe, a purely selective approach is generally less appropriate; in these cases, mucosal- and paracollicular-sparing strategies that preserve the peri-verumontanal region while allowing more complete lateral lobe enucleation may offer a more balanced compromise between ejaculation preservation and durable obstruction relief.

A similar balance applies to more complex mucosal- and paracollicular-sparing strategies (e.g., Double-n techniques). These approaches consistently improve ejaculatory outcomes compared with standard HoLEP, yet they are often associated with longer operative times and greater technical demands, which may limit broader applicability and could influence outcomes outside high-volume centers.

While ejaculation preservation has become an increasingly important patient-reported outcome, it must be balanced against operative efficiency, completeness of adenoma removal, and long-term durability. Several studies included in this review suggest potential trade-offs associated with EP-HoLEP, including longer operative times, reduced enucleated tissue weight, or higher retreatment rates in selected cohorts [18, 21, 22]. These findings indicate that ejaculation preservation may, in some cases, necessitate a narrower enucleation margin, reinforcing the need for careful patient selection and preoperative counselling.

Importantly, despite these trade-offs, most studies reported comparable short-term improvements in urinary symptom scores, maximum urinary flow rate, and continence outcomes between EP-HoLEP and standard HoLEP, consistent with broader HoLEP literature demonstrating the robustness of enucleation-based relief of obstruction [29, 30, 2]. In addition, refinements in HoLEP technique have been associated with improved perioperative recovery profiles—such as earlier catheter removal—supporting the broader feasibility of anatomically mindful modifications [28]. Ultimately, the choice of EP-HoLEP should be individualized through shared decision-making that explicitly balances anatomical feasibility, expected urinary benefit, durability of symptom relief, and the patient’s valuation of ejaculatory preservation. In addition, wider dissemination of HoLEP in general—and EP-HoLEP in particular—may be constrained in some settings by the need for specialized laser equipment, morcellation systems, and associated costs.

The overall quality of evidence is moderate but evolving. Although randomized and prospective comparative studies suggest a consistent signal favouring EP-HoLEP for preservation of AE, much of the available literature consists of single-centre studies with heterogeneous technique definitions, variable follow-up, and inconsistent assessment of sexual outcomes. Risk-of-bias assessment identified frequent concerns related to patient selection, deviations from intended interventions, and outcome measurement, particularly in retrospective cohorts. These limitations preclude definitive conclusions regarding the superiority of any single EP-HoLEP technique and highlight the need for standardized reporting. Taken together, these findings align with contemporary guideline emphasis on functional outcomes and patient-centred decision-making in BPH surgery.

Several limitations of this systematic review should be acknowledged. First, substantial heterogeneity in study design, surgical technique, and outcome definitions — particularly regarding ejaculatory outcomes — precluded quantitative meta-analysis. Second, definitions of ejaculation preservation varied across studies, and validated sexual function questionnaires were not uniformly employed. Many reports relied on subjective or non-standardized categorizations of ejaculation (e.g., ‘preserved,’ ‘reduced,’ or ‘absent’) rather than validated instruments such as the MSHQ-EjD-SF or structured semen analyses. This methodological variability likely introduces reporting bias and limits the reliability and comparability of ejaculatory outcomes across studies. Third, follow-up duration was limited in many reports, restricting assessment of long-term durability and retreatment risk. This review was intentionally limited to ejaculation-preserving modifications of HoLEP; therefore, other contemporary surgical techniques such as Aquablation or ThuLEP were not systematically evaluated. Although emerging data suggest favorable ejaculatory outcomes with various techniques in selected patients, direct comparisons with HoLEP remain limited and heterogeneous. Finally, most included studies originated from high-volume centres with significant HoLEP experience, which may limit generalizability to broader practice settings. These technically demanding modifications are also likely subject to a steeper learning curve, which may partly explain why most published series originate from high-volume centers.

In summary, the available evidence supports the anatomical plausibility and surgical feasibility of ejaculation preservation during HoLEP in selected patients. Rather than representing a single standardized procedure, EP-HoLEP encompasses a spectrum of anatomically informed adaptations aimed at minimizing disruption of peri-ejaculatory structures while retaining the benefits of enucleation. Future studies with standardized technique definitions, validated outcome measures, and longer follow-up are required to define the optimal balance between ejaculation preservation, functional efficacy, and long-term durability.

## Conclusions

Ejaculation-preserving modifications of HoLEP represent an evolving, anatomically grounded approach to the surgical management of benign prostatic hyperplasia in sexually active men. The available evidence suggests that selective preservation of peri-ejaculatory structures during enucleation can substantially improve rates of antegrade ejaculation without compromising short-term urinary functional outcomes in appropriately selected patients. Rather than constituting a single standardized technique, EP-HoLEP encompasses a spectrum of anatomically informed adaptations that differ in their dissection planes, extent of tissue preservation, and applicability across prostate morphologies. While these strategies appear feasible and clinically promising, the heterogeneity of techniques, outcome definitions, and follow-up duration limits definitive conclusions regarding long-term durability and optimal patient selection. Future prospective studies with standardized surgical definitions, validated sexual outcome measures, and longer follow-up are needed to define the most effective and durable ejaculation-preserving approaches within the HoLEP framework.

## Supplementary Information

Below is the link to the electronic supplementary material.

Supplementary Material 1

## Data Availability

All data generated or analyzed during this systematic review are included in the published article and its supplementary files, including extracted study characteristics and risk-of-bias assessments. No individual-level patient data were accessed.
