# Supplementary material for: Ejaculation-preserving holmium laser enucleation of the prostate: a systematic review of techniques, functional outcomes, and safety
Source: World J Urol. 2026 Mar 21;44(1):252. doi: 10.1007/s00345-026-06348-7 (PMC13005820; doi:10.1007/s00345-026-06348-7)
Supplement: Supplementary file 1 — Supplementary Material 1 [file 345_2026_6348_MOESM1_ESM.docx]

| **Supplementary Table 1.** Detailed search strategies for PubMed and Embase. | |  |
| --- | --- | --- |
| **PubMed** | ("Holmium"[Mesh] OR "Holmium laser" OR "HoLEP" OR "holmium laser enucleation of prostate" OR "holmium laser enucleation" OR "laser enucleation") AND ("Ejaculation"[Mesh] OR "ejaculation" OR "ejaculatory" OR "sparing" OR "preserving" OR "preservation" OR "sexual function") |  |
|  |  |  |
| **Embase** | ('holmium'/exp OR 'holmium laser' OR 'holep' OR 'holmium laser enucleation of prostate' OR 'holmium laser enucleation' OR 'laser enucleation') AND ('ejaculation'/exp OR 'ejaculation' OR 'ejaculatory' OR 'sparing' OR 'preserving' OR 'preservation' OR 'sexual function') |  |
|  |  |  |
